# A Comparison of Oxidative Stress Biomarkers in the Serum of Healthy Polish Dairy Goats with Those Naturally Infected with Small Ruminant Lentivirus in the Course of Lactation

**DOI:** 10.3390/ani11071945

**Published:** 2021-06-29

**Authors:** Natalia Kurhaluk, Halyna Tkachenko, Michał Czopowicz, Jacek Sikora, Daria M. Urbańska, Aldona Kawęcka, Jarosław Kaba, Emilia Bagnicka

**Affiliations:** 1 Department of Biology, Institute of Biology and Earth Sciences, Pomeranian University in Słupsk, 76-200 Słupsk, Poland; nataliakurhaluk@gmail.com (N.K.); halyna.tkachenko@apsl.edu.pl (H.T.); 2 Division of Veterinary Epidemiology and Economics, Institute of Veterinary Medicine, Warsaw University of Life Sciences, Nowoursynowska 159 C, 02-776 Warsaw, Poland; michal_czopowicz@sggw.edu.pl; 3 National Research Institute of Animal Production, 32-083 Balice, Poland; jacek.sikora@izoo.krakow.pl (J.S.); aldona.kawecka@iz.edu.pl (A.K.); 4 Institute of Genetics and Animal Biotechnology of the Polish Academy of Sciences, Postępu 36A, 05-552 Jastrzębiec, Poland; daria.m.urbanska@gmail.com

**Keywords:** oxidative stress biomarkers, Polish white improved goats, Polish fawn improved goats, SRLV

## Abstract

**Simple Summary:**

Many viruses, including human immunodeficiency virus 1, influenza virus, or Rift Valley fever virus, cause cell damage by generating reactive oxygen species and altering redox homeostasis. However, cells have developed various antioxidant mechanisms. We assumed that small ruminant lentivirus (SRLV), which has been found to infect sheep and goats worldwide can also disrupt the homeostasis of animals. SRLV target organs are the joints, lungs, brain, and the udder. To our knowledge, no information exists on the influence of SRLV infection on the oxidative processes occurring in goats. Understanding the influence of viral infection on oxidative stress may help develop novel antiviral treatments. Our study aimed to examine the effects of SRLV infection on oxidative stress biomarkers in the serum of dairy goats during lactation. No differences in any studied parameter at any stage of lactation were found between infected and uninfected goats. On the other hand, significant differences in almost all investigated parameters were found between stages of lactation, regardless of the infection status of goats. In conclusion, asymptomatic SRLV-infected goats do not reveal any apparent dysfunctions in serum oxidative stress biomarkers compared to their uninfected counterparts. The only changes in oxidative stress biomarkers observed during lactation appear to reflect the metabolic effort associated with milk production and developing pregnancy.

**Abstract:**

The present study examines the effects of natural infection by small ruminant lentivirus (SRLV) in the two most common goat breeds in Poland, i.e., Polish white improved and polish fawn improved. It focuses on biomarkers of oxidative stress, oxidatively modified proteins and antioxidant defenses, ceruloplasmin level as an acute phase protein, and the activities of antioxidant enzymes in the goat serum. It was conducted on 24 goats divided into two equal groups: one SRLV-seropositive (SRLV-SP) and another SRLV-seronegative (SRLV-SN). Both groups were identical in terms of breed and parity. Despite infection, the SRLV-SP goats demonstrated no symptoms of caprine arthritis-encephalitis. In addition, the SRLV-SP goats did not reveal pronounced dysfunctions in oxidative stress biomarkers in the serum compared to the SRLV-SN animals. However, both groups demonstrated elevated levels of the aldehydic and ketonic derivatives of oxidatively modified proteins during the lactation period. In addition, both groups retained a high total antioxidant capacity in serum despite the decrease of enzyme antioxidant defenses throughout the 200-day lactation period.

## 1. Introduction

The optimal level of reactive oxygen species (ROS) in the organism is controlled by the cellular antioxidant protection (AOP) system, comprising enzymatic and non-enzymatic elements. When the AOP system is insufficient, the organism is subjected to increasing levels of oxidative stress, resulting in a cascade of pathological processes, with extremely negative consequences for the organism. Viral infections are also associated with ROS generation and thus increased oxidative stress [1].

Many viruses have been demonstrated to cause cell damage by generating ROS and altering redox homeostasis [1,2,3]. Isaguliants et al. [2] reported a direct correlation between the ability of human immunodeficiency virus 1 (HIV-1) proteins, such as Tat and reverse transcriptase, to induce oxidative stress and their immunogenicity. In addition, infection by virulent influenza virus (IV) is characterized by heavy cellular infiltration and severe lung pathology, and both conditions are accompanied by oxidative stress and matrix metallopeptidase 9 (MMP-9) production [1]. *Flaviviridae* virus infections are also known to cause oxidative stress, affecting both the life cycle of the virus and the cellular metabolism. Furthermore, antiviral inflammatory signaling pathways are activated following infection [4]. Rift Valley fever virus (RVFV) infection leads to an increase in ROS production in liver cells due to the presence of the viral protein NSm (small cytosolic protein of RVFV, a major virulence factor in the mammalian host) in the mitochondria. Interestingly, the associated increase in cytokine and pro-apoptotic gene expression caused by infection was reversed with antioxidant treatment [5]. 

In addition, infection with dengue virus (DENV) leads to the accumulation of NADPH oxidase (NOX)-dependent intracellular ROS [6]. Conversely, a reduction of ROS level by chemical or genetic inhibition of the NOX complex weakens the innate immune responses to DENV infection and facilitates viral replication. ROS were also found to be essential in driving mitochondrial apoptosis in infected dendritic cells. ROS appear to play a critical role in stimulating the innate immune response to the virus and promoting apoptosis of human cells infected with DENV. Simultaneously, antioxidant pathways that are regulated by nuclear factor erythroid 2-related factor 2 (Nrf2) were activated to maintain redox homeostasis.

Because viruses cause an imbalance in the cellular redox environment, it can result in various responses depending on the virus and the cell type, including cell signaling, antioxidant defenses and reactive species generation. For example, ROS produced during cellular metabolism play an important role as signaling messengers, and can also stimulate inflammatory signaling pathways via protein kinases, transcription factors, and increased genomic expression of proinflammatory factors [7]. To counteract the oxidative effects deriving from the high chemical reactivity of ROS, and to maintain redox homeostasis, cells have evolved antioxidant mechanisms [8]. However, the presence of ROS is needed for the activation of cells providing antimicrobial immunity, i.e., neutrophils and macrophages, and the production of proinflammatory cytokines [7,8].

Although varying in their ability to induce ROS, viruses employ a common pathogenic pathway to defend against oxidative stress [4]. One such universal strategy employed by viruses to trigger antioxidant responses is the modulation of Nrf2 signaling [9]. 

The cellular antioxidant defense system acts under both physiological and pathological conditions to protect against the harmful actions of ROS and maintain cellular homeostasis. One component of the defense system comprises endogenously produced enzymatic antioxidants, the major ones being superoxide dismutase (SOD), catalase (CAT), and glutathione peroxidase (GPx) [8].

One virus that appears to disturb the homeostasis of goats is small ruminant lentivirus (SRLV), which has been found to infect many populations of sheep and goats worldwide [10,11,12]. Its wide prevalence makes it one of the most significant causes of decreased dairy goat production among small ruminants [10]. However, contradictory results have been obtained regarding milk yield and composition among infected goats [13,14,15,16]. The presence of the virus was first confirmed in the Polish population in 1996. This may have been due to the extensive import of Saanen and Alpine goats following 1990 [17]. 

The SRLV form a genetically diverse group, comprising five main genotype groups (A-E) and many more subtypes. Being in the *Retroviridae* family, SRLV integrates into the host DNA during replication. The virus causes caprine arthritis-encephalitis (CAE) and maedi-visna disease (MVD) in sheep [18]. Interspecies transmission has been confirmed [19]. Direct evidence of mixed infections with SRLV from four genetic groups has been found in both sheep and goats [20]. 

In the first stage of infection, SRLV infiltrates the dendritic cells of the respiratory tract, mucous membranes, or intestine. These cells migrate to the lymph nodes, where, in turn, monocytes are infected. The viral mRNA is transcribed into DNA to form a provirus, which is integrated into the host genome. Following this, the infected cells leave the lymph node and can cause systemic infection. Infection of bone marrow cells, myeloid stem cells, or myeloid precursors leads to persistent infection [21,22]. The target organs of infected monocytes are the joints, lungs [23], and the udder. In addition, in kids, the central nervous system can also be infected; however, this is extremely rare [10,24].

Information on the immune processes occurring in organisms infected with SRLV is still limited; however, some studies indicate that both innate and acquired immunities are involved in the host response [12,25,26]. However, to our knowledge, no information exists on the influence of SRLV infection on the oxidative processes taking place in goats. Understanding the influence of viral infection on oxidative stress in different stages of lactation may help to develop new antiviral treatments.

Under both physiological and pathological conditions, the cellular antioxidant defense system acts to protect against the harmful actions of ROS and maintain cellular homeostasis. One component of the defense system comprises endogenously produced enzymatic antioxidants, the major ones being superoxide dismutase (SOD), catalase (CAT), and glutathione peroxidase (GPx), as well as glutathione reductase (GR), and various proteins with pronounced antioxidative properties, such as ceruloplasmin [8]. The level of oxidative stress can be determined according to the level of lipoperoxidation based on the use of 2-thiobarbituric acid reactive substances (TBARS) as a marker.

Therefore, the aim of our study was to examine the effects of natural SRLV infection on selected biomarkers of oxidative stress, including TBARS, oxidatively modified proteins (protein carbonyl derivatives of amino acids, aldehyde derivatives, AD and ketone derivatives, KD) and antioxidant defenses (total antioxidant capacity (TAC), ceruloplasmin (Cp) level). The goats used in the study belong to the two most common breeds in Poland: Polish white improved (PWI) and Polish fawn improved (PFI).

## 2. Materials and Methods

### 2.1. Animals and Experimental Design

All procedures involving animals were performed according to the Guiding Principles for the Care and Use of Research Animals and were approved by the III Local Ethics Commission (Warsaw University of Life Science; Permission No. 31/2013).

The study was carried out throughout lactation in 24 dairy goats, selected from a herd of 50 does (Central Poland), belonging to two goat breeds: Polish white improved (PWI) and Polish fawn improved (PFI). The goats were fed in groups according to a system developed by the *Institute National de la Recherche Agronomique* (INRA) of France and adopted by the Research Institute of Animal Production (IZ PIB), Poland [27]. The basal diet consisted of maize silage and concentrates. Fresh hay was administrated *ad libitum* each afternoon. Water and saltlicks were also available *ad libitum*. The feed samples were analyzed for the content of basic components using standard methods [28]. The data on the ingredients and nutrient composition of the basal diet are shown by [29].

All goats in this herd were regularly tested serologically for the presence of antibodies against SRLV using a commercial ELISA test (ID Screen MVV/CAEV indirect screening test, IDVet Innovative Diagnostics, Grabels, France). Testing was performed twice a year, in June and December, for 20 years [30]. This testing was continued during the study period to eliminate any newly infected goats from the experimental group. The presence of the virus was also confirmed by isolation [20]. RT-qPCR analysis, performed according to Brinkhof et al. [31] in blood, found that the virus load was below detection levels, despite the presence of antibodies (Bagnicka—personal communication). This could explain the lack of observed symptoms of CAE in the infected goats.

The animals remained under the constant care of a veterinarian, who assessed the possible occurrence of infection and clinical signs of any other diseases. In addition, each goat was also clinically examined by certified specialists throughout the study (Diplomates of the European College of Small Ruminant Health Management—co-authors JK and MC). One group of animals used in the study were SRLV-seronegative (SRLV-SN, *n* = 12), i.e., free of SRLV antibodies in the blood, while the other group was SRLV-seropositive (SRLV-SP, *n* = 12) at least for two years before the start of observation. However, as it was mentioned above, all goats were asymptomatic, without any clinical sign of arthritis. All goats were between their third and eight lactations, with completed somatic development, to avoid any additive influences on homeostasis. The SRLV-SP and SRLY-SN groups were equally represented in terms of parity (3rd, 4th, and >4th), and breed (PWI vs. PFI). However, the only difference between these two breeds identified by transcriptomic analysis (~50 K) was found for the *Capra hircus* agouti signaling protein (ASIP), which is responsible for the coat color [31]. Moreover, in a study of the entire Polish active goat population, [32,33] did not report any differences between those two breeds in milk yield, fat, protein, or lactose content; however, they did indicate a higher somatic cell count in the milk of the PFI than the PWI goats. The animals were maintained in pens for 12 goats, and they remained in the herd at the end of the experiment and were not euthanized.

### 2.2. Samples

Blood samples were taken collected six times during lactation (the first sampling several hours after parturition) from the jugular vein into sterile 9 mL S-Monovette tubes with clot activator (Sarstedt, Nümbrecht, Germany). All sampling procedures were performed by a veterinarian. After centrifugation, the serum samples were removed and frozen at -20 °C and stored until analysis.

### 2.3. Biochemical Assays

*TBARS assay.* The level of lipid peroxidation was determined by quantifying the concentration of TBARS according to the method for determining the malondialdehyde (MDA) concentration [34]. This method is based on the reaction with thiobarbituric acid (TBA) in an acidic pH at 90–100 °C. In the TBA test reaction, MDA or MDA-like substances (produced during lipid peroxidation) and TBA react, with the production of a pink pigment with a 532 nm absorption maximum. 

*Protein carbonyl derivative assay.* The level of oxidative modified proteins (OMPs) was evaluated by the reaction of protein carbonyl derivatives (ketone-2,4-dinitro-phenylhydrazone) in serum with 2,4-dinitro-phenylhydrazine (DNFH). The rate of protein oxidative destruction was estimated as described by Zaitseva and Shandrenko [33]. DNFH was used to determine the carbonyl content in soluble and insoluble proteins. Carbonyl groups were determined spectrophotometrically based on the difference in absorbance at 370 (aldehydic derivatives, OMP_370_) and 430 nm (ketonic derivatives, OMP_430_).

*Assay of superoxide dismutase activity*. Superoxide dismutase (SOD) activity was assessed in an alkaline medium (pH 10.0) by its ability to dismutate superoxide produced during quercetin auto-oxidation as described by Kostiuk et al. [34]. The activity of SOD was expressed in units per mL of serum.

*Catalase activity assay*. Catalase (CAT) activity was determined as described by Koroliuk et al. [35]. Briefly, CAT activity was evaluated spectrophotometrically by measuring the decrease of H_2_O_2_ in the reaction mixture at the wavelength of 410 nm. One unit of CAT activity is defined as the amount of enzyme required for decomposition of 1 mmol H_2_O_2_ per min per L of serum.

*Measurement of glutathione reductase activity.* Glutathione reductase (GR) activity in the serum of goats was analyzed according to Glatzle et al. [36]. The GR activity was assayed spectrophotometrically by measuring NADPH_2_ reduction and was expressed as μmol NADPH_2_ per min per mL of serum.

*Assay of glutathione peroxidase activity.* Glutathione peroxidase (GPx) activity was determined according to Moin [37] by detecting the non-enzymatic utilization of GSH as reacting substrate. GPx activity was measured at an absorbance of 412 nm after incubation with 5,5-dithiobis-2-nitrobenzoic acid (DTNB). GPx activity was expressed as μmol GSH per min per mL of goat serum.

*Ceruloplasmin level assay*. The ceruloplasmin (Cp) level in the serum was assayed spectrophotometrically as described by Ravin [38] in 0.4 M sodium acetate buffer (pH 5.5), and 0.5% p-phenylenediamine at 540 nm. Ceruloplasmin was expressed in mg per L of serum.

*Measurement of total antioxidant capacity (TAC).* The TAC level in the serum was estimated spectrophotometrically according to Galaktionova et al. [39]. The TAC level was determined by measuring the TBARS level after ferrum/ascorbate induced by Tween 80 oxidation at 532 nm. The level of TAC in the sample (%) was calculated against the absorbance of the blank sample.

### 2.4. Statistical Analysis

Results are expressed as mean ± SEM (standard error). All variables were tested for a normal distribution using the Kolmogorov-Smirnov and Lilliefors tests (*p* > 0.05). The homogeneity of variance was checked using Levene’s test. The significance of the differences between the serum biomarkers of oxidative stress from the SRLV-SN and SRLV-SP goats was established by analysis of variance, with the model including the stage of lactation (1 to 6), the health state of the goats (SRLV-SN vs. SRLV-SP), breed (PWI vs. PFI), and number of lactation (3rd, 4th, or more than 4th). The ANOVA Friedman test and Kendall’s coefficient of concordance were also conducted with STATISTICA 13.3 software (Tibco Software Inc., Palo Alto, CA, USA). The significance of any differences between the SRLV-SN and SRLV-SP groups regarding the levels of lipid peroxidation, carbonyl derivatives, and antioxidant enzyme activities was examined using Student’s *t*-test. Differences were considered significant at *p* < 0.05 or *p* < 0.01 [40].

## 3. Results

### 3.1. Oxidative Stress

**Figure 1:**
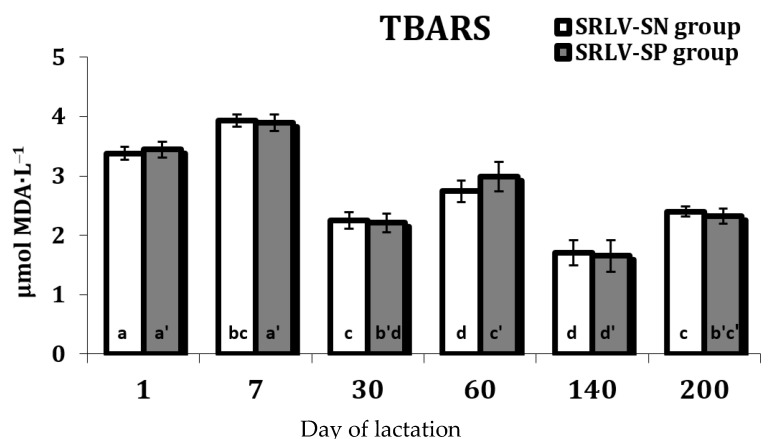
Lipid peroxidation, estimated by TBARS content (µmol MDA·L^−1^) in the serum of SRLV-SN and SRLV-SP goats during lactation. Values are expressed as means ± SEM; a, b, c, d—different letters indicate significant differences between stages of lactation within the SRLV-SN group at *p* < 0.05; a’, b’, c’, d’—different letters indicate significant differences between stages of lactation within the SRLV-SP group at *p* < 0.05; SRLV-SP—small ruminant lentivirus seropositive goats; SRLV-SN—small ruminant lentivirus seronegative goats.

**Figure 2:**
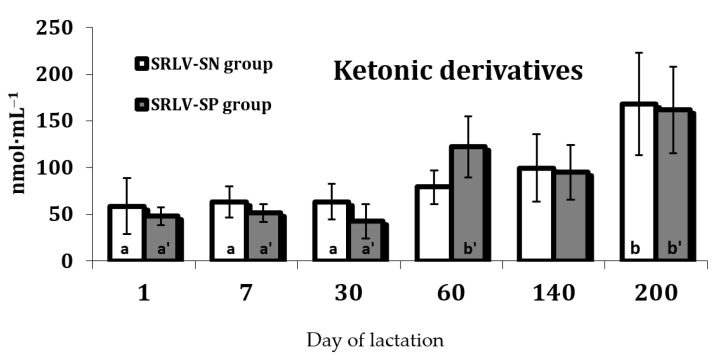
Ketonic derivatives of oxidatively modified proteins (nmol·mL^−1^) in the serum of SRLV-SN and SRLV-SP goats during lactation. Values are expressed as means ± SEM; a, b—different letters indicate significant differences between stages of lactation within the SRLV-SN group at *p* < 0.05; a’, b’—different letters indicate significant differences between stages of lactation within the SRLV-SP group at *p* < 0.05; SRLV-SP—small ruminant lentivirus seropositive goats; SRLV-SN—small ruminant lentivirus seronegative goats.

**Figure 3:**
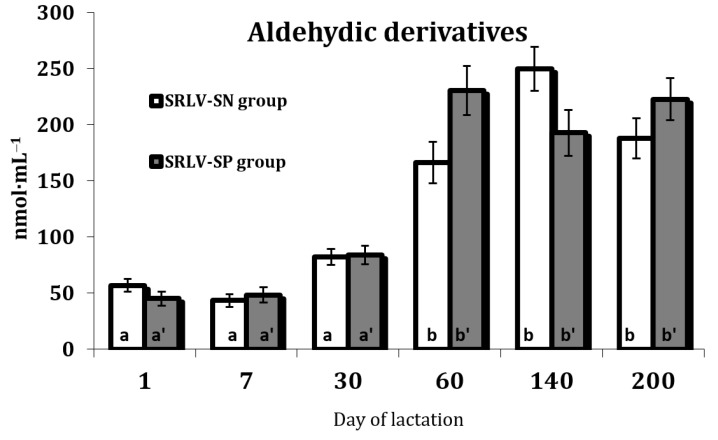
Aldehydic derivatives of oxidatively modified proteins (nmol·mL^−1^) in the serum of SRLV-SN and SRLV-SP goats during lactation. Values are expressed as means ± SEM; a, b—different letters indicate significant differences between stages of lactation within the SRLV-SN group at *p* < 0.05; a’, b’—different letters indicate significant differences between stages of lactation within the SRLV-SP group at *p* < 0.05; SRLV-SP—small ruminant lentivirus seropositive goats; SRLV-SN—small ruminant lentivirus seronegative goats.

**Figure 4:**
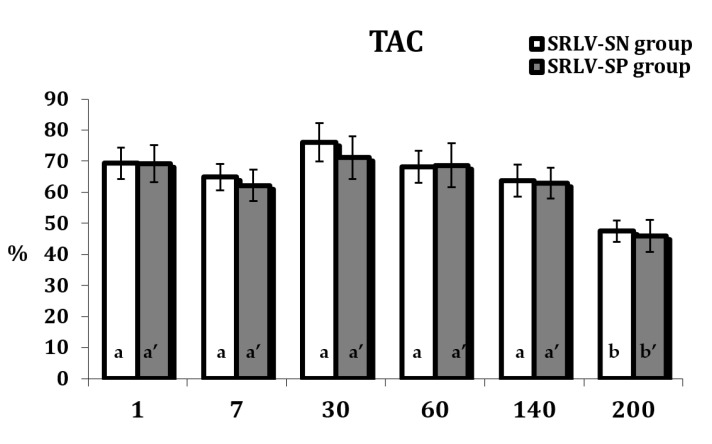

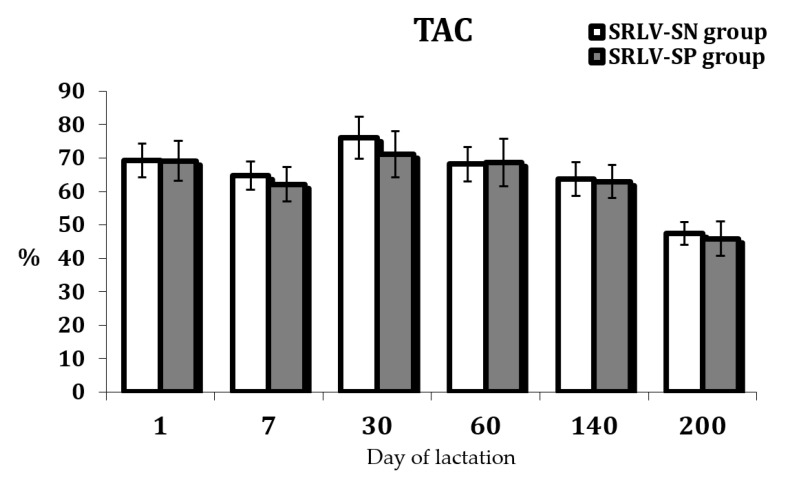
The total antioxidant capacity (TAC, %) in the serum of SRLV-SN and and SRLV-SP goats during lactation. Values are expressed as means ± SEM; a, b—different letters indicate significant differences between stages of lactation within the SRLV-SN group at *p* < 0.05; a’, b’—different letters indicate significant differences between stages of lactation within the SRLV-SP group at *p* < 0.05; SRLV-SP—small ruminant lentivirus seropositive goats; SRLV-SN—small ruminant lentivirus seronegative goats.

**Figure 5:**
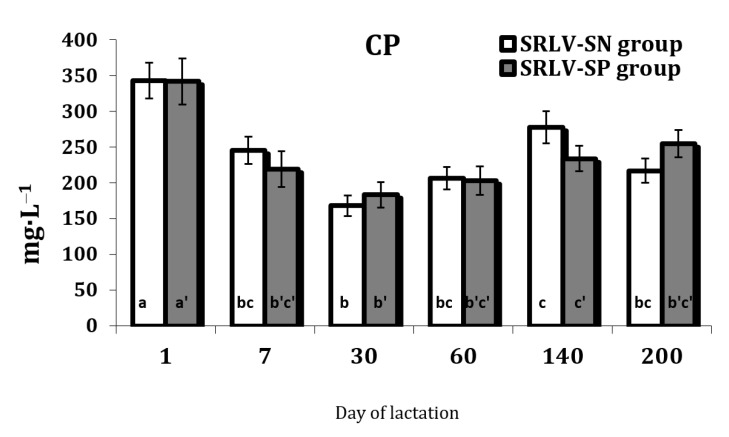
Ceruloplasmin level (Cp, mg·L^−1^) in the serum of SRLV-SN and SRLV-SP goats during lactation. Values are expressed as means ± SEM; a, b, c—different letters indicate significant differences between stages of lactation within the SRLV-SN group at *p* < 0.05; a’, b’, c’—different letters indicate significant differences between stages of lactation within the SRLV-SP group at *p* < 0.05; SRLV-SP—small ruminant lentivirus seropositive goats; SRLV-SN—small ruminant lentivirus seronegative goats.

**Figure 6:**
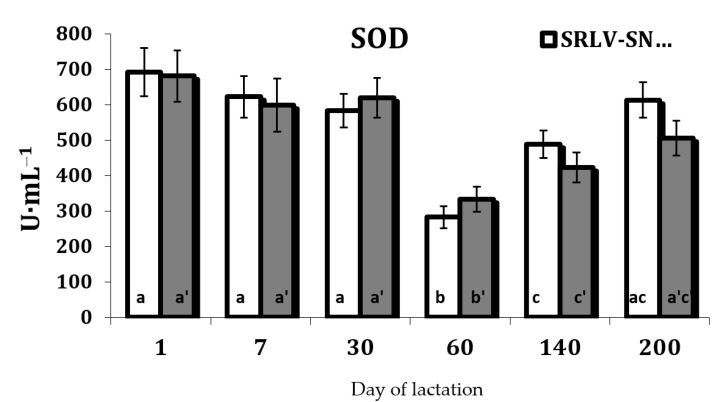
Superoxide dismutase activity (SOD, U·mL^−1^) in the serum of SRLV-SN and SRLV-SP goats during lactation. Values are expressed as means ± SEM; a, b, c—different letters indicate significant differences between stages of lactation within the SRLV-SN group at *p* < 0.05; a’, b’, c’—different letters indicate significant differences between stages of lactation within the SRLV-SP group at *p* < 0.05; SRLV-SP—small ruminant lentivirus seropositive goats; SRLV-SN—small ruminant lentivirus seronegative goats.

**Figure 7:**
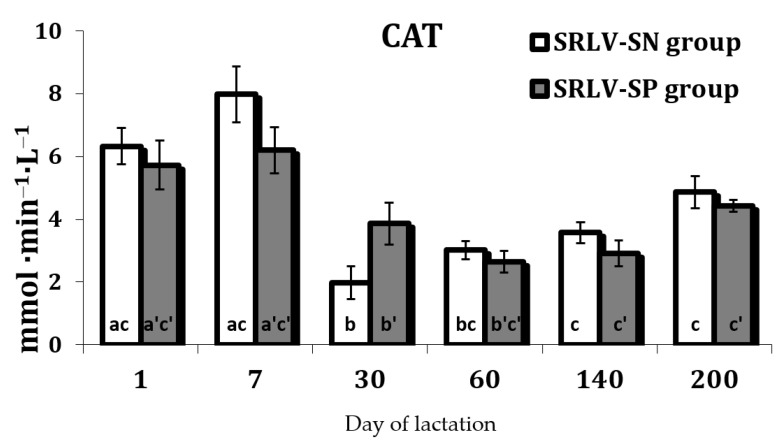
Catalase activity (CAT, mmol H_2_O_2_·min^−1^·L^−1^) in the serum of SRLV-SN and SRLV-SP goats during lactation. Values are expressed as means ± SEM; a, b, c—different letters indicate significant differences between stages of lactation within the SRLV-SN group at *p* < 0.05; a’, b’, c’—different letters indicate significant differences between stages of lactation within the SRLV-SP group at *p* < 0.05; SRLV-SP—small ruminant lentivirus seropositive goats; SRLV-SN—small ruminant lentivirus seronegative goats.

**Figure 8:**
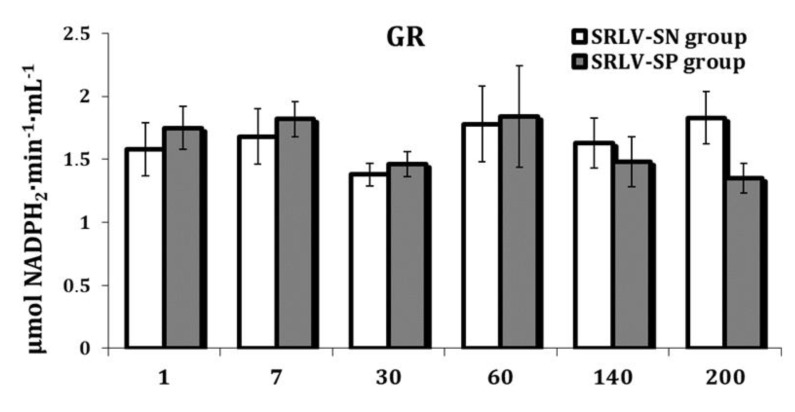
Glutathione reductase activity (GR, μmol NADPH_2_·min^−1·^mL^−1^) in the serum of SRLV-SN and SRLV-SP goats during lactation. Values are expressed as means ± SEM; SRLV-SP—small ruminant lentivirus seropositive goats; SRLV-SN—small ruminant lentivirus seronegative goats.

**Figure 9:**
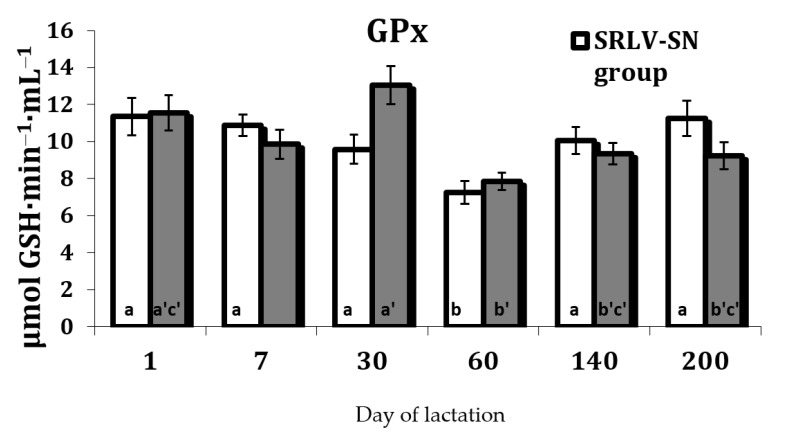
Glutathione peroxidase activity (μmol GSH·min^−1^·mL^−1^) in the serum of SRLV-SN and SRLV-SP goats during lactation. Values are expressed as means ± SEM; a, b, c—different letters indicate significant differences between stages of lactation within the SRLV-SN group at *p* < 0.05; a’, b’, c’—different letters indicate significant differences between stages of lactation within the SRLV-SP group at *p* < 0.05; SRLV-SP—small ruminant lentivirus seropositive goats; SRLV-SN—small ruminant lentivirus seronegative goats.

No differences were found between SRLV-SN and SRLV-SN goats for any studied parameter at any studied stage of lactation (Figure 1, Figure 2, Figure 3, Figure 4, Figure 5, Figure 6, Figure 7, Figure 8 and Figure 9); however, differences were found between stages of lactation for almost all studied parameters. In addition, the direction of the changes was the same in both the SRLV-SN and SRLV-SP groups (Table S1).

The level of TBARS, i.e., the by-product of free radical-induced lipid peroxidation, in the serum of SRLV-SP (*p* < 0.000) and SRLV-SN (*p* = 0.000) goats during lactation (on days 1, 7, 30, 60, 140, and 200) is presented in Figure 1. In both groups, the highest level of TBARS on was observed on day 7 after delivery, with a lower level observed on day 1 and the lowest on day 140. No differences between groups were observed in any stage of lactation.

No differences in the levels of either OMP, i.e., aldehydic and ketonic derivatives, were observed between the two groups (*p* > 0.05). Nevertheless, OMP levels were influenced by stage of lactation (*p* < 0.01) (Table S1), namely their levels were found to increase over the course of lactation.The highest levels of the ketonic derivates were observed on day 60 (only RLV-SP group) and day 200 of lactation, while aldehydic derivatives peaked on day 140 (only SRLV-SN group) (*p* < 0.05), with similar tendencies observed in both groups (Figure 2 and Figure 3). 

The stage of lactation also influenced TAC (Table S1). A significant decrease in TAC was observed at the end of lactation compared to day 1 for both groups (*p* < 0.05) (Figure 4).

Significant differences in serum Cp concentration were observed between the stages of lactation in both the SRLV-SN (*p* = 0.000) and SRLV-SP goats (*p* = 0.023) (Table S1). The changes occurred in parallel for both groups. Cp concentration was the lowest on day 30 after parturition compared to day 1 (Figure 5). 

### 3.2. Antioxidant Enzyme Activity

Serum SOD was influenced by the stage of lactation in both SRLV-SN (*p* = 0.000) and SRLV-SP goats (*p* = 0.004) (Table S1). In both groups, SOD activity was lowest on day 60 (peak of the lactation). This level then increased on day 140 and was fully restored on day 200 compared to day 1 (Figure 6).

Both groups demonstrated similar changes in CAT activity during lactation (SRLV-SN *p* = 0.010; SRLV-SP *p* = 0.015) (Table S1). The highest activity was observed until day 7 after parturition. CAT then underwent only small changes between early lactation (day 30) and peak (day 60), full lactation (day 140), and the end (day 200) of lactation as compare to the first day (Figure 7).

Serum GR activity remained stable in both SRLV-SN (*p* = 0.252) and SRLV-SP goats during lactation (*p* = 0.227) (Table S1, Figure 8).

The serum GPx activity was also influenced by the stage of lactation (Table S1). In the SRLV-SN goats, the activity was lowest on day 60 of lactation. This value then increased and remained stable until day 200 (*p* < 0.05). In the SRLV-SP goats, activity was high at the first stages of lactation, peaking on day 30 after parturition (*p* < 0.05), then falling almost 2-fold by day 60 and remaining stable until day 200 (Figure 9); however, no differences were found between groups.

## 4. Discussion

The SRLV-SP and SRLY-SN groups were equally represented in terms of parity (3rd, 4th, and >4th) and breed (PWI vs. PFI). However, in our earlier studies, the only difference between these two breeds identified by transcriptomic analysis (~50 K) was found for the *Capra hircus* agouti signaling protein (ASIP), which is responsible for the coat color [41]. Moreover, in a study of the entire Polish active goat population, Bagnicka et al. [42,43] did not report any differences between those two breeds in milk yield, fat, protein, or lactose content; however, they did indicate a higher somatic cell count in the milk of the PFI than the PWI goats.

ROS and reactive nitrogen species (RNS) are byproducts of aerobic metabolism in various cell compartments, including the mitochondria and ER. They play key roles in the maintenance of redox homeostasis during both normal physiological functions, and in numerous pathophysiological states [4,44]. ROS generation can cause various cellular consequences depending on their overall concentration at steady-state levels and on their site of generation [45]. Unbalanced ROS production and scavenging contributes to oxidative stress. Despite having a negative influence on health, this stress can also be used to treat clinical conditions, such as cancer, with a certain degree of clinical success [46]. 

As a natural defense system, the cell regularly produces antioxidants, or adapts other mechanisms, to eliminate the ROS and thus maintain the balance of antioxidation and oxidation processes [4]. ROS and RNS overproduction can result in various destructive effects by disrupting the antioxidant defenses and impairing cellular integrity and functionality [47]. In vivo, free radicals are primarily responsible for chemical modifications and damage to proteins (aggregation and denaturation), lipids (peroxidation), and carbohydrates. They also induce changes to the DNA structure, leading to mutations or cytotoxic effects [48].

Very little, if anything, is currently known about the relationship between known biomarkers and oxidative stress in SRLV-SP goats. The most significant finding of the present study is that the SRLV-SP and SRLV-SN goats appear to demonstrate no difference in the levels of oxidative stress biomarkers. However, it should be stressed that throughout the period of infection, the SRLV-SP goats were asymptomatic for CAE, as well as other diseases, including mastitis. This suggests that animals without clinical symptoms of CAE, with low virus loads, do not suffer any oxidative stress and their welfare is not disturbed. 

However, as oxidative stress is related to a number of aspects of the pathogenesis of viral etiologic agents, this was a surprising finding. Viruses affect the cellular redox balance by increasing oxidants, such as superoxide and nitric oxide, and inhibiting the synthesis of antioxidant enzymes, such as SOD, CAT, and GPx [8]. A study of SRLV by Mdurvwa et al. [49] found, in contrast to our results, that the antioxidant potential of serum from SRLV-infected goats of various age groups demonstrated significantly higher catalase activity than serum from healthy controls. In addition, this activity increased over time following infection with SRLV. However, similar to our results, no differences in total SOD or GPx activity were observed, although Cu, Zn-SOD levels were elevated in the infected goats. The authors also reported a positive correlation between serum catalase activity and hydrogen peroxide (H_2_O_2_) scavenging activity [49]. Although a transient increase was observed in lactate dehydrogenase (LDH), no correlation was observed between increased serum catalase activity and LDH activity. The authors also note decreased oxyradical production in SRLV-infected goats. This may be due to the increase in serum catalase, a scavenger of endogenous free radicals, such as H_2_O_2_. Nevertheless, Santos et al. [50] showed that, as in the present study, no pronounced dysfunctions in blood or milk polymorphonuclear leukocytes or in monocytes/macrophages were observed in naturally SRLV-infected goats.

However, some changes in oxidative biomarkers have been observed in other viral diseases. For example, Balikci et al. [51] compared blood oxidative stress biomarkers (MDA and nitric oxide), antioxidant levels (SOD and GPx), and acute-phase proteins activity (haptoglobin and serum amyloid A) between aborting and non-aborting goats with border disease (BD) caused by border disease virus (BDV). Both the infected non-aborting and infected aborting groups demonstrated a decrease in GPx and SOD activities and an increase in MDA, NO, haptoglobin, and serum amyloid A levels compared to the non-infected group. Additionally, the aborting goats displayed significantly higher MDA, NO, haptoglobin, and serum amyloid A levels, and lower SOD levels than the non-aborting groups. 

The oxidant-antioxidant balance has also been found to be disrupted in sheep with *peste des petits ruminants* (PPR), which in turn can cause further oxidative damage. Nisbet et al. [52] examined the changes in the biomarkers of free oxygen radicals and antioxidant activity, i.e., MDA, GPx, and SOD, in sheep with PPR. Their findings indicate that the PPR-positive group demonstrated a significantly higher mean MDA level and lower mean GPx and SOD activities compared to controls.

Our present findings are, in general, in agreement with the those in our previous study on cytokine and acute-phase protein (APP) gene expression [25,26], at both the mRNA and protein level, in the blood cells/sera of SRLV-SP but CAE-asymptomatic and SRLV-SN goats. Although Reczyńska et al. [26] found a higher concentration of serum amyloid A (SAA) in blood serum of SRLV-SP goats, this may also indicate that viral multiplication was promoted, since this APP may inhibit antibody production and could stimulate the differentiation of monocytes to macrophages, an essential step in viral multiplication. In addition, Jarczak et al. [25] report a decreased concentration of the pro-inflammatory cytokines IL-1α, IL-6, and INF-β in the sera of SRLV-SP goats. Both these studies indicate that the systemic immune system of infected goats is impaired, thus preventing them from fighting the disease. 

As it was stressed above, those findings are consistent with the results of the present study—SRLV appears to avoid the systemic immune system and does not cause oxidative stress. They are also consistent with Tian et al. [53], who note that the concentration of cytokines undergoes a series of changes under prolonged stress in humans. Constant stress continues to increase the pro-inflammatory cytokines, which finally cause inflammation and may lead to various diseases. This may mean that the animals involved in the study are in the early stage of the stress caused by infection. All of our studies, both the present study and previous ones, were conducted on asymptomatic goats. 

Even so, the levels of several oxidative stress biomarkers changed over the course of lactation. In dairy cows, oxidative stress has been closely studied during the transition period and early lactation [54,55,56], during which, a loss of overall antioxidant potential can be observed. This phenomenon is connected with increased metabolic demands and leads to a weakening of the antioxidant defense of dairy cows, accompanied by a greater risk of metabolic diseases [55]. In addition, Piccione et al. [57]. reported that oxidative processes increase at the end of lactation in sheep. 

Of the various markers of protein oxidation, the best studied is protein carbonyl formation, which increases in tissues and organs during oxidative stress [58,59]. In our study, the levels of both aldehydic and ketonic derivatives of oxidatively modified proteins underwent some fluctuations during lactation. The aldehydic derivatives increased from a low level at the beginning of lactation to peak on day 140 in SRLV-SN goats, while the ketonic derivates peaked on day 200 in both groups (Figure 2 and Figure 3). However, some studies suggest that the accumulation of protein oxidation products increases with age [58]. The increase in the level of ketonic derivatives observed on day 60 of lactation in the SRLV-SP goats is probably associated with the peak of lactation, as well as gaps between nutrient supply and demand. Lactating animals use their own energy reserves to cover their demand as the dietary intake alone is insufficient. The fact that a high level of ketonic derivates was only observed in the serum of SRLV-SP goats may mean that the burden for an infected organism was greatest during the highest milk production period. Under these conditions (i.e., day 60 of lactation in SRLV-SP goats), the energetic metabolism becomes destabilized: the energy loss exceeds the physiological capacity of the animal’s feed intake. 

ROS-induced activation of lipid peroxidation, occurring in response to hormonal changes during the lactation process, significantly intensifies oxidative modifications in proteins, observed in the accumulation of aldehydic and ketonic derivatives. On the other hand, the serum level of ketonic derivatives peaked on day 200 in both groups, which may indicate that the udder was being prepared for drying off. This period is very critical for all high-yielding dairy animals, including goats. This first stage of the lactation process is associated with an increase in milk yield; however, in the same periods of lactation, increased levels of both aldehydic and ketonic derivatives were observed (Figure 2 and Figure 3). Free radical-induced oxidative stress and intensification of protein metabolism results in oxidative modification of proteins because the goat organism does not receive the necessary amount of energy from the feed and uses the energy stored in the body. 

During the lactation period, serum TBARS level was found to decrease in both SVRL-SN and SVRL-SP goats after 30 days and remained stable for the rest of the study (Figure 1). High levels of lipid peroxidation were observed on day 1 and day 7 of the experiment, and these can be associated with the physiological changes in the organism of goats related to the postpartum period. This might also be associated with the transition period between colostrum and milk production, which also places a considerable burden on the animal, mainly due to the rapid increase in milk production. The TBARS level was slightly lower on day 60, but still high. This is probably connected with the peak of lactation, i.e., the highest milk production. In contrast, full lactation i.e., on day 140 and day 200, was associated with low TBARS levels, and this was probably related to the physiological stabilization of the lactation process. In goats, changes in metabolism can decrease milk production, thus probably allowing animals with significant energy loads to cover their needs related to lactation.

TAC level remained high throughout lactation; however, a decrease was observed on day 200 (Figure 4). This reduction is probably associated with the initiation of the drying off process. Under these conditions, the free radical processes and antioxidant defenses become destabilized. The accumulation of aldehydic and ketonic derivatives of oxidatively modified proteins, together with the depletion of antioxidant defenses, causes a significant decrease in TAC, and increase of oxidative stress. The observed changes may reflect the dysfunction of the body under these conditions.

The changes of the antioxidant defenses observed under these conditions in both SRLV-SN and SVRL-SP goats during lactation may reflect changes in the level of oxidative stress biomarkers. This increase in stress, resulting from an increase in the production of free radicals, may induce the activation of the antioxidant defense in tissues. Our present findings reveal a decrease in the activities of antioxidant enzymes (SOD, CAT, and GPx) after 60 days of lactation (Figure 6, Figure 7 and Figure 9). However, in contrast to our results, several studies indicate that the activities of SOD and GPx were reduced in some viral infections. Nisbet et al. [52] highlight that GPx and SOD activities were significantly lower in sheep with *peste des petits ruminants* than uninfected controls. Some clinical studies also report lower CAT and SOD activities in the blood of hepatitis B virus-infected patients [60]. 

However, SRLV infection does not appear to influence the activities of antioxidant enzymes in the asymptomatic stage of the disease. Then again, they were found to be influenced by the stage of lactation. Increased levels of protein damage biomarkers can lead to changes in SOD, CAT, and GPx activities. Their reduction suggests that the antioxidant defenses remain inadequate, probably due to increased oxidative-induced protein modification. The decrease in antioxidant enzyme activities observed in the current study may be due to oxidative damage occurring in the cellular structures as a result of the inability to fully detoxify free radicals. As SOD, CAT, and GPx are involved in the conversion of radicals into less effective metabolites, these changes, coupled with an increase in the levels aldehydic and ketonic derivatives of oxidatively modified proteins, confirm the presence of oxidative stress during the lactation period, both in the SRLV-SN and SVRL-SP goats.

Ceruloplasmin (Cp) has been shown to exhibit antioxidant functions, which have a beneficial effect under several pathological conditions [61,62]. Cp catalyzes the oxidation of ferrous (Fe^2+^) into ferric ions (Fe^3+^), with the process involving four of the sic copper atoms associated with Cp. Dioxygen can also act as an electron acceptor in the absence of any insufficiently reduced ROS, such as O^2−^ or H_2_O_2_. This can also determine the binding of iron to transferrin and ferritin [63]. Pro-inflammatory agonists of the acute phase reaction, such as IL-6 and TNF-α, increase hepatic cell Cp synthesis by a transcriptional mechanism [64]. Cp may act as an antioxidant and as an acute-phase reactant [65,66]. Some Cp regulation was observed to the end of the current study; however, although no complete recovery of Cp level was observed by day 200, compared to the first day after parturition, the final level was the same as that on day 7 (Figure 4). The elevated Cp level may have manifested as a response to systemic inflammation [66] and its high level just after parturition is connected probably with the high burden associated with delivery. Cp level was found to decrease on day 30 of lactation, indicating that the perinatal period is over. Therefore, a key finding of our study is that pronounced elements of oxidative stress appear to be absent from infected but asymptomatic animals. We propose this as an area for further research, by including symptomatic animals or animals with a very high viral load in studies. This will help identify the primary links between the effects of viral infection on biomarkers of oxidative stress and the mechanisms initiated by the virus under these conditions.

## 5. Conclusions

In conclusion, the goats naturally infected with SRLV but without clinical sign of CAE did not reveal any pronounced dysfunctions in serum oxidative stress biomarkers compared to uninfected animals. The only changes in oxidative stress biomarkers observed during lactation probably reflect the burden of goat metabolism caused by perinatal or drying off periods, or milk production. The two groups demonstrated similar biomarkers of oxidative stress in each stage of lactation, which may mean that SRLV does not trigger the immune system and does not cause oxidative stress in all SRLV-SP goats, but the asymptomatic ones. The next step of the study will be to examine samples derived from goats with clear clinical CAE symptoms and detectable virus loads.

## Data Availability

The datasets used and/or analyzed during the current study are available from the corresponding author on reasonable request.

## Supplementary Materials

The following are available online at https://www.mdpi.com/article/10.3390/ani11071945/s1, Table S1: The ANOVA Friedman test and Kendall’s coefficient of concordance of the serum biomarkers of oxidative stress in the SRLV-SN and SRLV-SP goats with stage of lactation as fixed effect.
