# Supplementary material for: A Comparison of Oxidative Stress Biomarkers in the Serum of Healthy Polish Dairy Goats with Those Naturally Infected with Small Ruminant Lentivirus in the Course of Lactation
Source: Animals (Basel). 2021 Jun 29;11(7):1945. doi: 10.3390/ani11071945 (PMC8300365; doi:10.3390/ani11071945)
Supplement: Supplementary file 1 [file animals-11-01945-s001.zip › animals-1224767-supplementary.pdf]

Supplementary file:

Table S1. The ANOVA Friedman test and Kendall's coefficient of concordance of the serum biomarkers of oxidative stress in the SRLV-SN and SRLV-SP goats with stage of lactation as fixed effect.

Parameter	SRVR-SN			SRVR-SP		
	χ^2 ANOVA	τ-Kendall	p value	χ^2 ANOVA	τ-Kendall	p value
TBARS	52.59	0.49	0.000	33.72	0.42	0.000
OMP AD	21.81	0.73	0.001	38.75	0.52	0.000
OMP KD	15.70	0.78	0.008	32.19	0.64	0.000
TAC	40.81	0.51	0.000	70.19	0.56	0.000
CP	24.90	0.83	0.000	13.00	0.65	0.023
SOD	11.57	0.58	0.000	16.90	0.56	0.004
CAT	9.14	0.45	0.010	14.09	0.47	0.015
GR	6.60	0.26	0.252	6.91	0.28	0.227
GPx	11.28	0.75	0.046	10.27	0.35	0.012

TBARS - Thiobarbituric acid reactive substances; OMP AD - oxidative modified proteins aldehyde derivatives; OMP KD – oxidative modified proteins ketone derivatives; TAC – total antioxidant capacity; Cp – ceruloplasmin; SOD - superoxide dismutase; CAT – catalase; GR – glutathione reductase; GPx – glutathione peroxidase; SRLV-SN – SRLV-seronegative goats; SRLV-SP – SRLV-seropositive goats.
